# On the Importance of Nanoparticle Necks and Carbon Impurities for Charge Trapping in TiO_2_

**DOI:** 10.1021/acs.jpcc.3c00430

**Published:** 2023-05-03

**Authors:** Michael J. Elser, Ellie Neige, Thomas Berger, Mario Chiesa, Elio Giamello, Keith McKenna, Thomas Risse, Oliver Diwald

**Affiliations:** † Institute of Particle Technology (LFG), Friedrich-Alexander-Universität Erlangen-Nürnberg, Cauerstraße 4, Erlangen 91058, Germany; ‡ Department of Chemistry and Physics of Materials, Paris-Lodron Universität Salzburg, Jakob-Haringerstrasse 2a, Salzburg 5020, Austria; § Department of Chemistry and NIS Centre, University of Torino, via Giuria 7, Torino I-10125, Italy; ∥ School of Physics, Engineering and Technology, University of York, Heslington, York YO10 5DD, United Kingdom; ⊥ Institut für Chemie und Biochemie, Freie Universität Berlin, Arnimallee 22, Berlin 14195, Germany

## Abstract

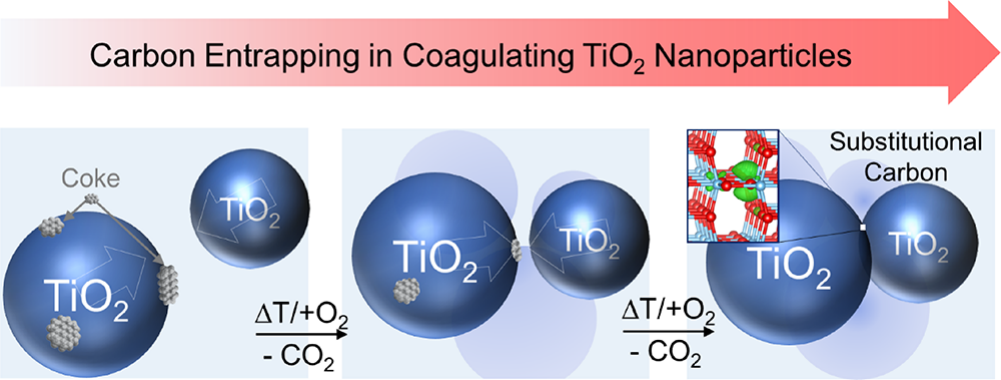

Particle attachment and neck formation inside TiO_2_ nanoparticle networks determine materials performance in sensing, photo-electrochemistry, and catalysis. Nanoparticle necks can feature point defects with potential impact on the separation and recombination of photogenerated charges. Here, we investigated with electron paramagnetic resonance a point defect that traps electrons and predominantly forms in aggregated TiO_2_ nanoparticle systems. The associated paramagnetic center resonates in the *g* factor range between *g* = 2.0018 and 2.0028. Structure characterization and electron paramagnetic resonance data suggest that during materials processing, the paramagnetic electron center accumulates in the region of nanoparticle necks, where O_2_ adsorption and condensation can occur at cryogenic temperatures. Complementary density functional theory calculations reveal that residual carbon atoms, which potentially originate from synthesis, can substitute oxygen ions in the anionic sublattice, where they trap one or two electrons that mainly localize at the carbon. Their emergence upon particle neck formation is explained by the synthesis- and/or processing-induced particle attachment and aggregation facilitating carbon atom incorporation into the lattice. This study represents a substantial advance in linking dopants, point defects, and their spectroscopic fingerprints to microstructural features of oxide nanomaterials.

## Introduction

Point defects play a key role in functional metal oxides and there is a continuous need to increase our understanding of their formation, their stability, and their influence on the materials properties.^1−5^ Apart from their importance for mass transport and sintering, point defects in intergranular regions can serve as trapping sites to promote charge carrier recombination and/or to retard transport of charge carriers for their collection in energy conversion processes. At a larger observation scale than point defects, solid–solid interfaces that correspond to particle necks are important structural features inside nanoparticle-based ensembles. Interparticle necking was shown to have a substantial impact for catalytic, photocatalytic, photoelectronic, and electronic applications.^3−7^ The exploration of potential interconnections between morphological features, the abundance of point defects, and spectroscopic fingerprints^8−12^ represents a major challenge in defect engineering in functional materials.

In oxygen-deficient and electronically reduced titanium dioxide (TiO_2–*x*_), the excess spin density is distributed over the titanium cations surrounding the defect,^13−15^ which corresponds to the electronic reduction of Ti^4+^ to Ti^3+^ ions. In terms of a charge compensation mechanism, this differs from those of paramagnetic defects in other metal oxides where the excess electron density is localized at the vacancy site^16^ comparable to F^+^ centers in insulators of pronounced ionicity, such as in alkali halides or alkaline earth oxides.^11,17^

In the materials science community, there seems to be agreement that the electronic structure of the impurity-free TiO_2_ polymorphs rutile and anatase do not provide sufficient stabilization for electron trapping inside the center of the vacancy.^18,19^ On the other hand, symmetrical EPR signals with *g* factors in the range between the free spin value *g* = 2.0023 and *g* = 2.004 were directly assigned in a number of reports and without significant proofs to single electron oxygen vacancy centers in TiO_2_.^20−24^ Authors of related studies connect their observations to the early work of Serwicka et al.,^25−27^ who found that the adsorption of electron acceptors such as O_2_, SO_2_, or SF_6_ on oxygen-deficient TiO_2_ increases the intensity of an isotropic signal at *g* = 2.003. She attributed this phenomenon to the adsorption-induced localization of conduction band electrons at oxygen vacancy sites. Later studies by other groups even suggested a correlation between this defect and photocatalytic activity of TiO_2_ in the range of visible light.^20,28^ A recent defect characterization study on mesoporous and nanocrystalline TiO_2_ nanoparticle networks^29^ reports an interesting charge/discharge behavior that occurs upon materials exposure to slow electrons. Related materials, which provide trapping sites for long-lived charge carriers – electrons and holes at room temperature and in the dark – were found to exhibit enhanced activity for photocatalytic CO_2_ reduction.^29^

A series of studies related to the design of photocatalysts have focused on the impact of carbon admixture to the TiO_2_ lattice in a concentration range between impurity doping,^30^ to oxycarbides, and finally to titanium carbides TiC.^31,32^ Some of these studies also included EPR data and reported the presence of the abovementioned EPR resonance^21,24,33−36^ without directly connecting the origin of the EPR center to carbon.

A direct linkage between the abundance of spectroscopically accessible point defects that participate in electron trapping, charge carrier recombination and altered surface reactivity,^28,37^ and microstructural nanoparticle arrangements is a key issue in materials design. The present study will explore the correspondence between the abundance of the abovementioned paramagnetic sites and the structural and microstructural powder properties of polycrystalline TiO_2_ materials as their hosts. From previous investigations, we learned that independent synthetic pathways can induce particle aggregation and particle necking and – at the same time – favor the formation of these paramagnetic point defects.^1^ Moreover, another focus of this study is to revisit the adsorption-induced formation and/or intensity enhancement of EPR signals close to the free electron *g* value. On TiO_2_, such effects have previously been associated with a localization of conduction band electrons involving electron centers in the surface and/or near surface region of polycrystalline TiO_2_.^25−27^ We address the question of whether localization of conduction band electrons at specific defects or the direct interaction between isolated paramagnetic defects with O_2_ can explain these phenomena.

The structure of the paper is as follows: first, we present results on TiO_2_ nanoparticle samples synthesized by flame spray pyrolysis (FSP), which exhibit significant concentrations of particle–particle interfaces and necks and exhibit a paramagnetic electron center with a signal around the free electron *g* value. Motivated by this, we review materials synthesis and processing approaches that lead to enhanced concentrations of particle necks and solid–solid interfaces hosting such defects. In the following section, we describe and analyze results from a detailed EPR investigation of the electron center. Supported by density functional theory (DFT) calculations that explore the paramagnetic properties of substitutional carbon atoms inside the TiO_2_ anatase lattice in detail, we can now rationalize the emergence of these trapping sites in networks of interconnected TiO_2_ nanocrystals. An alternative explanation for the previously reported adsorption-induced localization of conduction band electrons will be presented.^25−27^

## Experimental Section

### Gas Phase Synthesis of TiO_2_ Nanoparticles

TiO_2_ nanoparticles were synthesized either by metal–organic chemical vapor synthesis (MOCVS) or by flame spray pyrolysis (FSP):

#### Metal–Organic Chemical Vapor Synthesis (MOCVS)

The MOCVS reactor consists of a fused silica tube placed inside a cylindrical furnace and a preheating zone to evaporate the precursor (Ti(IV) isopropoxide, Sigma-Aldrich, 99.999%) at *T* = 393 K.^38^ Argon gas (5.0) transports the gaseous precursor from the preheating zone into the furnace, where decomposition occurs at 1073 K. Stable process conditions are guaranteed by the spatial separation of the precursor evaporation and the reaction zone. Continuous pumping keeps the residence time of resulting nuclei within the reactor short and prevents substantial coarsening and coalescence.

#### Flame Spray Pyrolysis (FSP)

A precursor solution of titanium tetra isopropoxide (TTIP, Sigma-Aldrich, 97%) in toluene solution was prepared (anhydrous, Sigma-Aldrich, 99.8%). The solution was then injected by a syringe pump with a flow rate of 2 mL·min^–1^ into the nozzle of the flame burner and atomized by oxygen as dispersion gas. Further process details are described in refs (39) and (40).

The as-synthesized nanoparticle powder was annealed in oxygen and vacuum to remove carbon remnants and residual water from the particle surfaces. Heating rates, dwell times, and environmental gas atmospheres were as follows: first, the powder was annealed under high vacuum conditions to 873 K using a heating rate of 10 K·min^–1^. This temperature was held for 60 min under continuous pumping *p* < 10^–5^ mbar. Finally, 20 mbar of oxygen was introduced for 30 min followed by evacuation for an additional 30 min. The oxygen admission–evacuation cycle was repeated two times. After a final oxygen admission step, the sample was cooled down in an oxygen atmosphere to *T* < 493 K in order to achieve a stoichiometric composition inside the TiO_2_ nanoparticle powder.

#### Materials Characterization by XRD and TEM

X-ray diffraction (XRD) measurements were performed on a Bruker AXS D8 Advance diffractometer using Cu Kα radiation (λ = 154 pm). Crystalline domain sizes *d*_XRD_ were determined from powder diffraction data using the Debye–Scherrer equation. Transmission electron microscopy (TEM) data were obtained using a JEOL JEM-F200 cold field emission transmission electron microscope (Jeol Ltd., Tokyo, Japan) operating at 200 kV. Images were recorded using a TVIPS F216 2k by 2k CMOS camera (TVIPS GmbH, Gauting, Germany) and the samples were measured on lacey carbon grids coated with copper.

#### Electron Paramagnetic Resonance

**Figure 1:**
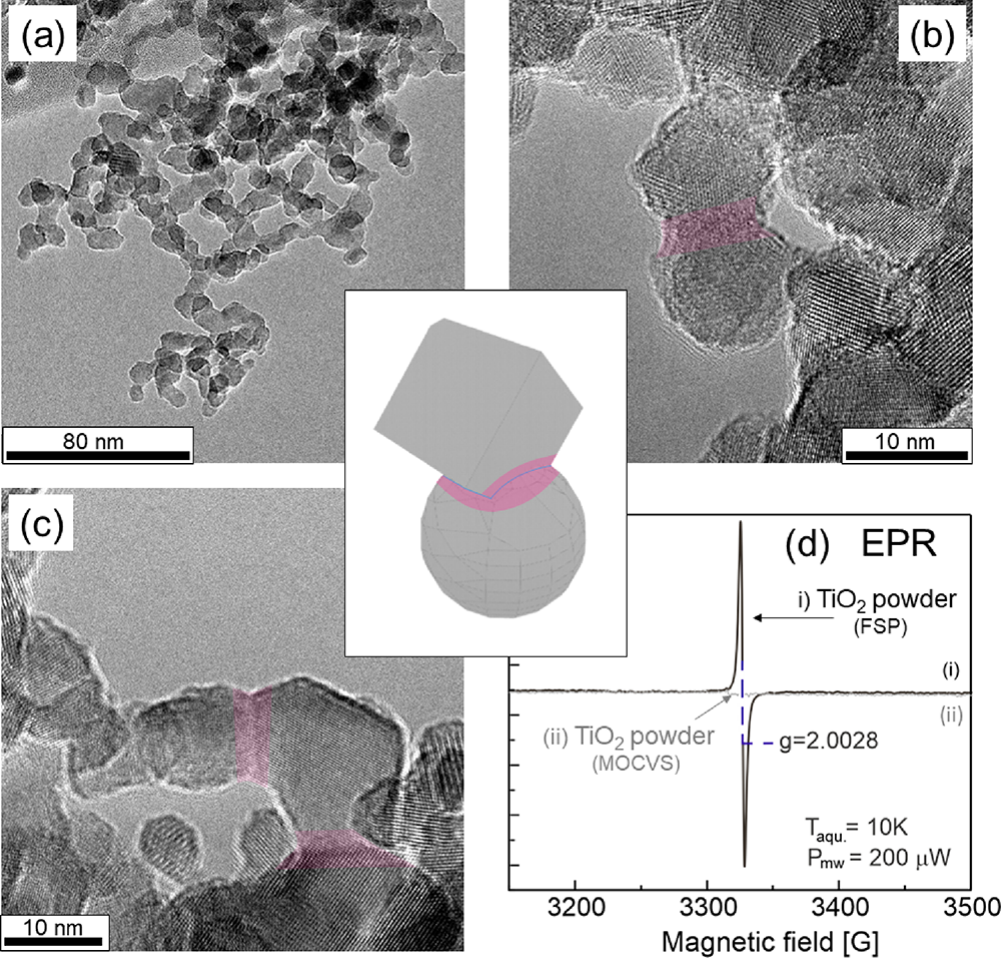
TEM images (a–c) and representative EPR spectrum (d) related to TiO_2_ nanoparticle powders that were synthesized by flame spray pyrolysis (FSP). The representative EPR spectra in panel (d) relate to oxidized FSP grown material (i) in comparison to that synthesized by metal organic chemical vapor synthesis (MOCVS) (ii).^1,38^ Different from the MOCVS nanoparticle powders, FSP powders show high concentrations of necks between nanoparticles (highlighted in panels (b) and (c)). The EPR spectra in panel (d) were acquired on nanoparticle powders after identical oxygen treatment protocols at 873 K. Only for the FSP grown powder we measured an isotropic signal at *g* = 2.0028.

For EPR measurements, the powder sample was inside a Suprasil quartz glass tube connected to an appropriate high vacuum pumping system (*p* < 10^–6^ mbar). Spectra acquired on MOCVS and FSP grown TiO_2_ nanoparticle powders (Figure 1) were performed with a Bruker EMXplus-10/12/P/L X-band spectrometer equipped with a waveguide Cryogen-Free System from Oxford Instruments. The spectra were recorded at 10 K with a field modulation frequency of 100 kHz, modulation amplitude of 0.2 mT, and microwave frequency of 9.30 GHz. Spin quantification was carried out with the Xenon software from Bruker. The detailed EPR analysis discussed along Figures 3, 5, and 6 was carried out with a Bruker EMX 10/12 spectrometer using a Bruker ER 4102ST standard rectangular resonant cavity in the TE102 mode. The *g* values were determined on the basis of a DPPH standard. For quantitative measurements, the spin concentrations were obtained by double integration of EPR signals, which were measured at *T* = 77 K using a microwave power of 200 μW.

#### Density Functional Theory Calculations

DFT calculations for bulk anatase TiO_2_ with and without carbon defects were performed using the CP2K simulation package and the truncated PBE0 hybrid-DFT exchange–correlation functional PBE0-TR-LRC.^41^ This functional has been parameterized to minimize self-interaction errors in the description of localized electron and hole polarons by variation of the percentage of Hartree–Fock exchange (α) to ensure that Koopmans’ condition (a necessary requirement for an exact functional) is satisfied.^42^ The optimized functional for anatase (α = 11.5%) predicts optimized lattice constants of *a* = 3.795 Å, *c* = 9.607 Å and a single electron band gap of 2.93 eV in good agreement with available experimental data,^18,30^ and more generally the generalized Koopmans’ condition approach has been shown to yield extremely good densities and quasiparticle energy gaps when compared against exact solution of the many-electron Schrödinger equation for simple model systems.^43^ More details on the approach for parameterization are given in refs (42) and (43). Triple ζ basis sets were used for titanium, oxygen, and carbon^44,45^ and the Goedecker–Teter–Hutter pseudopotentials available within CP2K.^46−48^ The plane wave energy cutoff, a reference grid that controls the Gaussian mapping onto the multigrid, is set to 60 Ry. Five multigrids are used for mapping products of Gaussians onto a real-space integration grid with a cutoff of 600 Ry for the finest level of the multigrid. The electronic convergence was set to 1 × 10^–6^ Ry per self-consistent field (SCF) cycle, and the Broyden–Fletcher–Goldfarb–Shanno method is used for optimization of cell vectors and bulk geometries until forces were below 8 × 10^–4^ Ry/*a*_0_ (0.02 eV/Å). The use of the auxiliary density matrix method reduces the computational cost of the expensive Hartree–Fock integrals permitting calculations on very large supercells. A 5 × 5 × 2 (600 atoms) anatase supercell with dimensions 18.977 × 18.977 × 19.214 Å was used for modeling a single carbon substituting an oxygen site defect (C_O_). The supercell structure was optimized at constant volume for *q* = 0, −1, and −2 e charge states of the defect. The one-electron energy levels were referenced to the bulk valence band edges to produce electronic structure diagrams for the defects. The structure and spin density of the C_O_^–1^ defect was visualized using the VESTA program.^49^

## Results and Discussion

Flame spray pyrolysis (FSP) is a well-suited synthesis technique for the production of TiO_2_ nanoparticles.^39,50−55^ In agreement with other studies,^56^ the particle systems that were synthesized here are characterized by uniform and narrow particle size distributions, and the average values coincide with those of the average crystallite domain size as derived from XRD reflection broadening and application of the Scherrer equation (Supporting Information, Figures S1 and S2). After controlled oxidation treatment of the nanoparticle powders, the 13 nm primary particles form stable aggregates and – as evidenced in the TEM micrographs of Figure 1a–c – host significant concentrations of solid–solid interfaces and necks between the particles.

TiO_2_ anatase nanoparticles from metal organic chemical vapor synthesis (MOCVS) show comparable crystallinity, particle size (Figures S1 and S2, Supporting Information), and morphology.^55^ After powder synthesis and processing, the resulting particle ensembles form soft aggregates (agglomerates) of more open structure, where no evidence for incomplete particle fusion and necking between different particles was obtained by TEM.^38^ This is in contrast to the FSP derived material (Figure 1a–c). Apart from other fundamental FSP powder properties (crystallinity, particle size, and morphology) that are comparable to those of the MOCVS derived powders (Supporting Information, Figures S1 and S2), we identified an intense EPR signal at *g* = 2.0028 for the FSP material (Figure 1d, (i) black trace), that is absent in the spectra of the MOCVS derived powder (Figure 1d, (ii) gray trace) despite identical parameters for materials processing. The signal saturation characteristics and temperature dependence are identical to those for strongly aggregated nanoparticle systems^1^ and will be described in detail below.

### Approaches to Generate Hard TiO_2_ Nanoparticle Aggregates with Particle Necks

**Figure 2:**
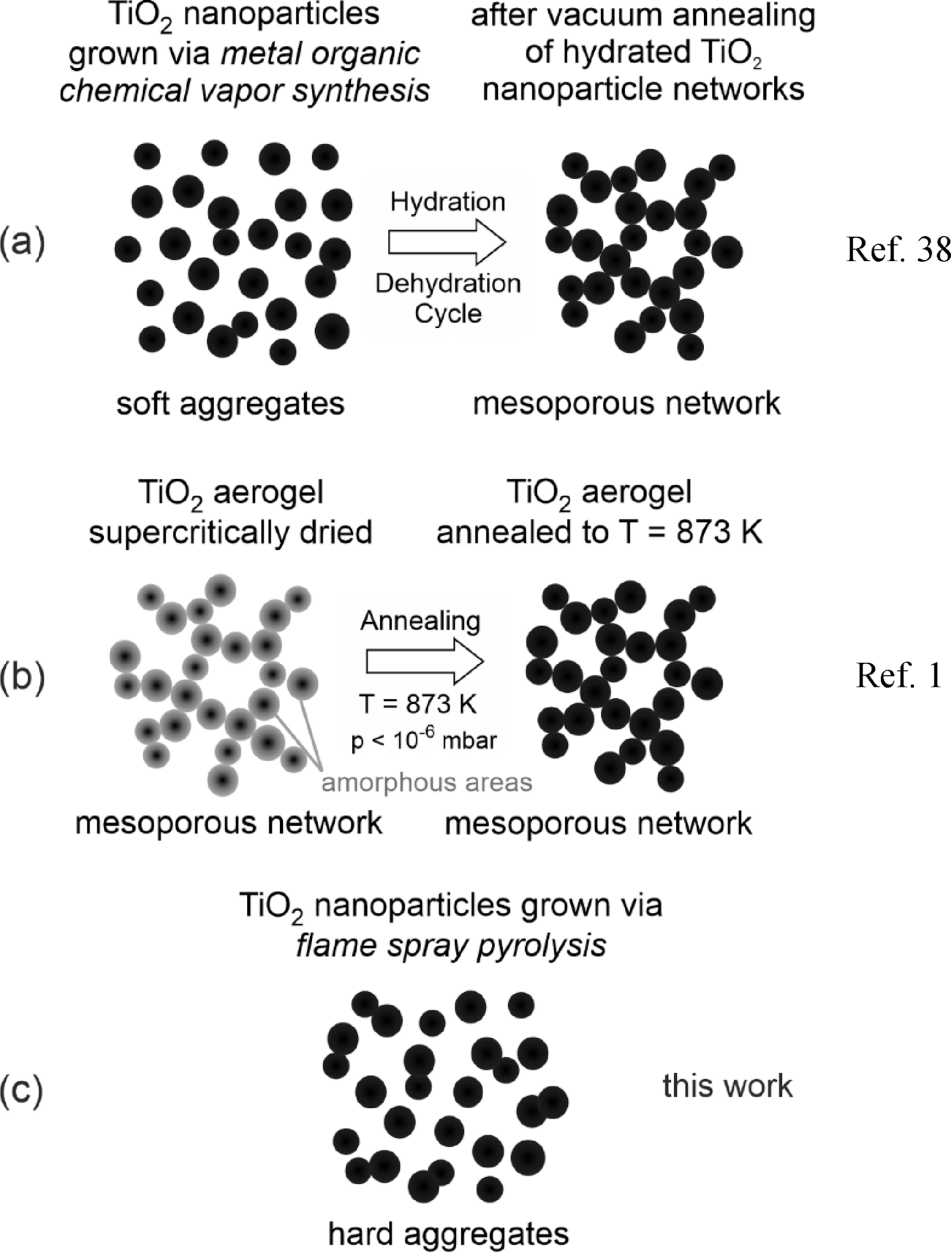
Scheme illustrating different approaches to generate TiO_2_ nanoparticle powders and nanoparticle networks with enhanced concentrations of particle–particle interfaces and necks.

Figure 2 summarizes different synthetic approaches to either generate continuous TiO_2_ nanoparticle networks made up from primary anatase nanocrystals with diameters of less than 15 nm (a and b) or nanocrystal powders where larger concentrations of particle necks result from synthesis and processing. Samples that were produced via metal organic chemical vapor deposition (MOCVS) correspond to powders of loosely agglomerated crystalline particles.^57^ The particles aggregate and form solid–solid interfaces when brought into contact with condensed water and subjected to a hydration–dehydration cycle with a final annealing step in vacuum (*p* < 10^–5^ mbar).^1,38^

Alternatively, sol–gel processing of ethanediolato(titanate) (EGMT) for TiO_2_ aerogel synthesis induces nucleation and growth of particles as nanocrystalline nodes that are interconnected via amorphous necks and bridges. Thermal annealing at *T* = 873 K completes crystallization at the expense of the amorphous moieties (Figure 2b). As reported in detail elsewhere,^1^ processes (a) and (b) lead to nano- and microstructures that strongly resemble each other in terms of their primary particle size, crystallinity, and aggregate structures.

Pore size distributions of annealed TiO_2_ aerogels (Figure 2b) and hydrated TiO_2_ nanoparticle networks (Figure 2a) together with electron microscopy and XRD data^1,38^ point to mesoporous nanoparticle networks with a high abundance of particle–particle interfaces and necking regions between the individual particles. Although extended hard particle aggregates, where most of the particles are connected to their neighbors via solid–solid interfaces, i.e., necks (Figure 2a,b), do not exist in FSP grown materials (Figures 1a–c and 2c), in such materials, we found considerable concentrations of particle dimers and trimers that again exhibit particle necking as a result of incomplete particle fusion during particle growth in the flame.

We now turn to the paramagnetic particle properties of materials systems derived from the three approaches a, b, and c in Figure 2. We exemplify this for a mesoporous nanoparticle network derived from metal organic chemical vapor synthesis (MOCVS). Particle aggregation and formation of an electronically reduced mesoporous nanoparticle network was achieved by applying a hydration–dehydration cycle to dry nanoparticle powders followed by vacuum annealing to 873 K (see scheme of Figure 2a).

**Figure 3:**
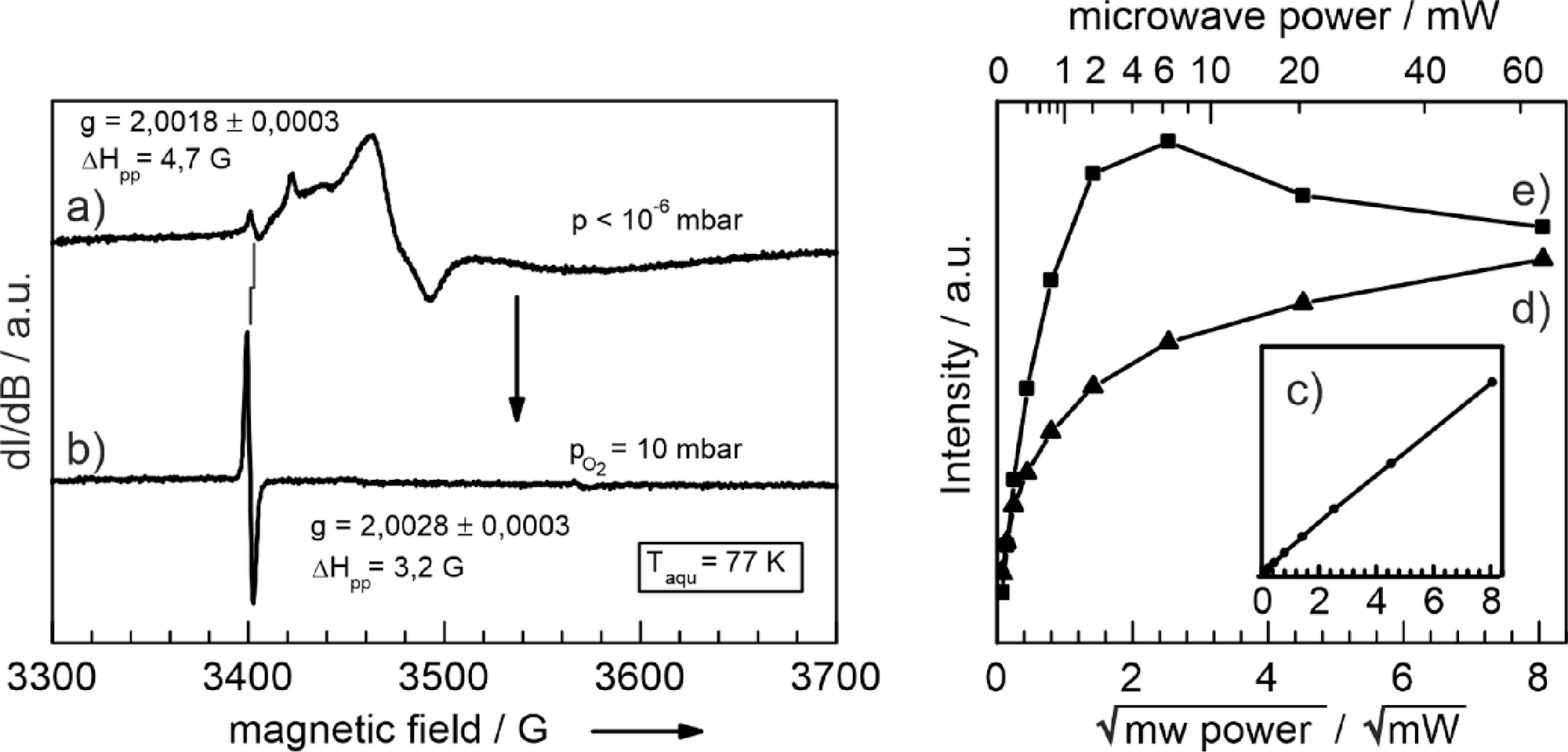
Left: X-band CW-EPR spectra of vacuum annealed (i.e., reduced) TiO_2–*x*_ nanoparticle networks and recorded at 77 K. These were measured either (a) under dynamic vacuum or (b) in an O_2_ atmosphere after room temperature admission of 10 mbar O_2_ from the gas line connected to the spectrometer system to the sample cell. Right: microwave power saturation plots (*T*_aqu_ = 77 K) for the signals presented in the left panel; (c) Ti^3+^ ions (from spectrum a); (d) EPR center at *g* = 2.0018 in vacuum (spectrum a); (e) EPR center at *g* = 2.0028 generated and measured in the presence of adsorbed O_2_ (spectrum b).

In the EPR spectrum of a reduced TiO_2–*x*_ nanoparticle network, the high magnetic field range (Figure 3a) shows a complex signal envelope that is specific to Ti^3+^ (d^1^) ions, the electronic and geometric structure of which was previously analyzed in great detail.^8,58,59^ There is an additional weak signal at *g* = 2.0018, which is typically observed in particle systems that are rich in solid–solid interfaces.^1^ Conversely, it has not been detected in dry processed MOCVS TiO_2_ powders, which are made up of loosely agglomerated particles. While the Ti^3+^ species do not show any saturation effect in the microwave power range 1 ≤ *P*_MW_ ≤ 60 mW (Figure 3c), the isotropic signal at *g* = 2.0018 (Figure 3d) saturates at *P*_MW_ > 2 mW (Figure 3e).

Adsorbed oxygen has three major effects on the EPR properties of TiO_2–*x*_ nanoparticle networks (Figure 3b): (i) O_2_ bleaches the Ti^3+^ specific resonances, (ii) shifts the resonance position of the isotropic signal from *g* = 2.0018 to 2.0028, and (iii) gives rise to a significant signal intensity enhancement of the latter signal.

**Figure 4:**
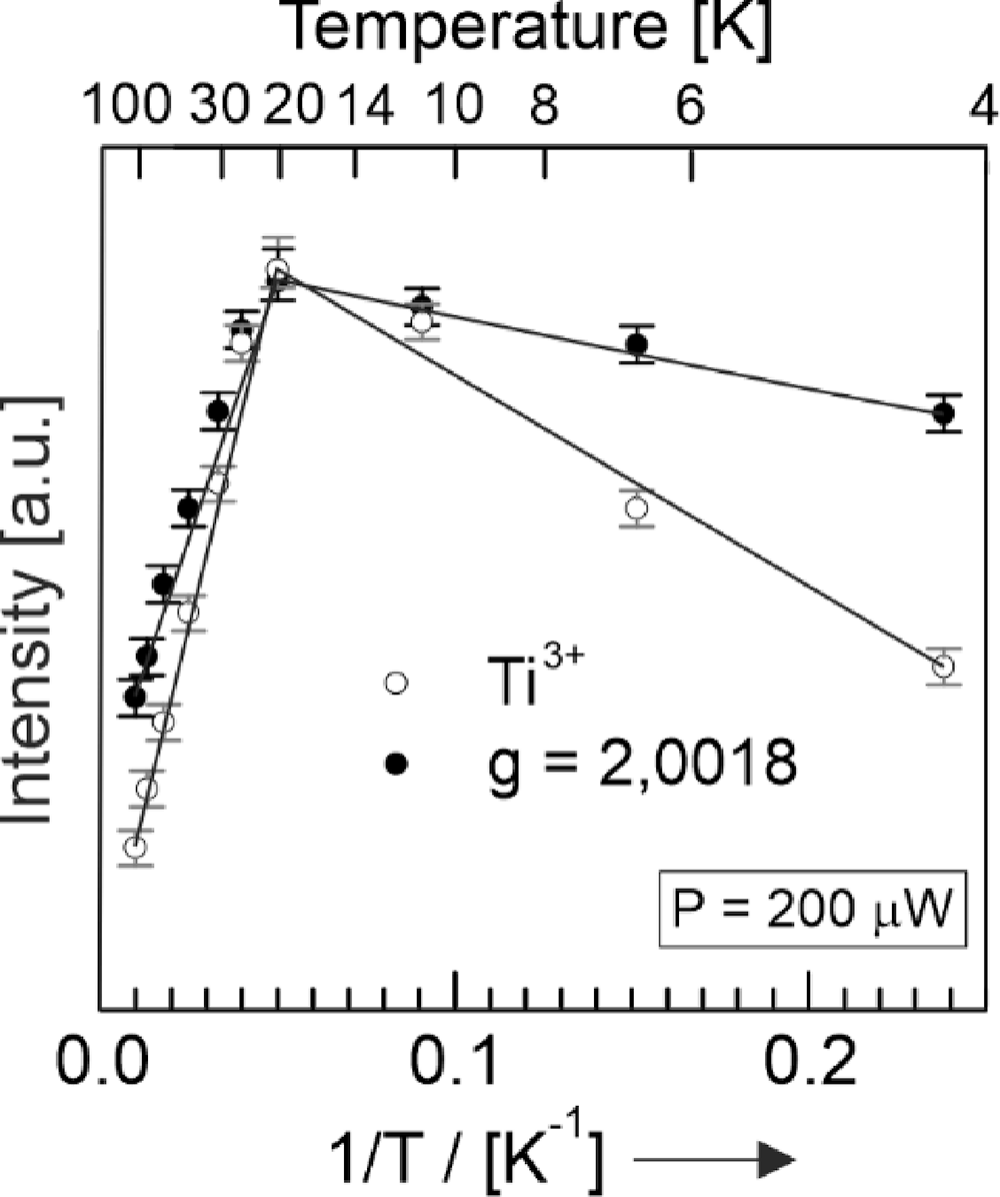
*T* dependence of the EPR signal intensities related to Ti^3+^ sites (open circles) and the electron center at *g* = 2.0018 (full circles) in vacuum annealed (i.e., reduced) TiO_2–*x*_ nanoparticle networks (see also Figure 3a). The integral intensity values for *T* = 20 K were normalized for the sake of comparison. Please note that the Ti^3+^-related EPR signal intensities are at least one order of magnitude larger than those related to the electron center.

Figure 4 shows the intensities of the two EPR signals as a function of inverse temperature for the vacuum annealed TiO_2–*x*_ sample (Figure 3a) in the range between 4 and 100 K (Figure 4). With constant linewidth and signal shape, the two signals exhibit the intensity trends plotted in Figure 4a,b.

Between 100 and 20 K, the intensities of both signals increase linearly with inverse temperature as expected by the Curie law (Figure 4). Hence, above 20 K, the corresponding species behave as isolated and non-interacting paramagnetic entities. Upon further cooling down to 4 K, the shape and peak-to-peak widths of the EPR signals for the electron center and the Ti^3+^ ions do not change. However, their intensities decrease, which is unexpected for paramagnetic species. From previous studies of slightly oxygen-deficient TiO_2_, we learned that in the nanocrystalline form, such systems exhibit a significant fraction of EPR-silent excess electrons.^60,61^ Based on the available data, it is not possible to provide a conclusive explanation for the intensity decrease of the signals below 20 K, but the coinciding maxima for the two chemically different species at 20 K and with no indication for spatial proximity point to a long-ranged coupling of these species, which may involve the abovementioned excess electrons. Additional magnetic interactions cannot be excluded and a more detailed explanation requires additional investigations.

**Figure 5:**
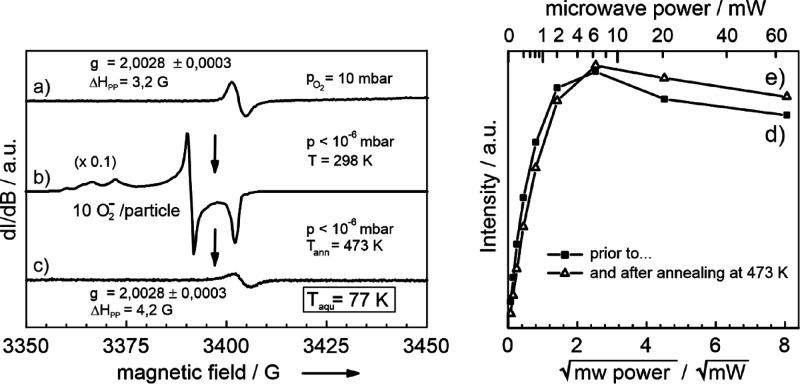
CW X-band EPR spectrum showing the electron center signal (a) after O_2_ admission to a vacuum annealed (i.e., reduced) TiO_2–*x*_ anatase nanoparticle network. Room-temperature evacuation eliminates spin exchange interactions of molecular oxygen with surface adsorbed O_2_^–^ resulting in intense and characteristic resonances in the EPR spectrum (b). Annealing to *T* = 473 K decomposes the O_2_^–^-related resonances but does not alter the relaxation properties of the spin center resonating at *g* = 2.0028 (curves e and d). Both microwave saturation curves were achieved with an initial O_2_ pressure of 10 mbar at room temperature and subsequent cooling to *T* = 77 K.

We now turn to the effect of oxygen admission to vacuum-annealed TiO_2_ nanoparticle surfaces, which can be divided along the following three major lines (Figure 3b):

i) When electronically reduced surface and subsurface sites – such as the Ti^3+^ ions – emerge in TiO_2–*x*_ upon vacuum-annealing, adsorbed O_2_ as an electron acceptor drives an interfacial electron transfer to transform into anionic surface oxygen species that can be paramagnetic (O_2_^–^) or diamagnetic (O_2_^2–^, O^2–^).
ii) As a triplet molecule O_2_ undergoes spin exchange interaction with other paramagnetic surface species (radicals and point defects), this leads to broadening of the EPR signal related to these species and, ultimately, to its extinction at enhanced local O_2_ coverages. Pumping off the molecularly adsorbed oxygen from the sample cell at room temperature reverses the effect and re-establishes the EPR signal. Related phenomena are well-suited to indicate whether paramagnetic states are located at the surface or subsurface region.
  For the specific case here, the effective spin exchange interaction between adsorbed triplet oxygen and the paramagnetic O_2_^–^ also explains why the latter signal is not measured in the presence of adsorbed oxygen (Figure 3b). It, however, appears upon pumping (see below Figure 5, a → b).
iii) O_2_ adsorption can alter the local electronic and geometric structure of surface or subsurface defects resulting in a change of resonance position (*g* value) as well as the spin–lattice relaxation times.

We investigated the effect of oxygen admission on the paramagnetic samples’ properties. For this purpose, we added 10 mbar O_2_ gas at room temperature, sealed the cell, and cooled the sample down to an acquisition temperature of *T*_aqu_ = 77 K. This process shifts the resonance position of the isotropic signal from *g* = 2.0018 to *g* = 2.0028 being associated with a reduction of the peak-to-peak line width (Figures 3b and 5a). Furthermore, a significant intensity enhancement of the signal is observed. As in the absence of oxygen (Figure 3d), the isotropic signal at *g* = 2.0028 saturates at microwave powers *P*_MW_ > 2 mW (Figure 3e). In addition, O_2_ bleaches the Ti^3+^-specific resonances (Figure 3b).

From the gas volumes determined for the different cell compartments and the defined O_2_ pressure (10 mbar) inside the cell at room temperature, we calculated the amount of adsorbed oxygen. Assuming a uniform adsorbate distribution over the high surface area material (∼30 mg TiO_2_, A = 3.9 m^2^ from the specific surface area of 130 m^2^ · g^–1^), this corresponds to 0.4 monolayer equivalents (0.4 ML) if all O_2_ molecules are adsorbed at *T* = 77 K. The effect of a condensing O_2_ gas on the EPR spectrum of surface bound radicals at low temperature becomes evident by comparing the situation discussed above to one where the amount of O_2_ gas was reduced by evacuating the cell. The resulting EPR spectrum obtained after subsequent cooling to 77 K is shown in Figure 5b. The spectrum is dominated by signals associated with O_2_^–^ species adsorbed at surface Ti^4+^ ions as adsorption sites. The intensity of these species is so large that it is impossible to identify possible contributions of the species with an EPR signal at *g* = 2.0028 (Figure 5a). Thus, admission of molecular oxygen to a reduced TiO_2–*x*_ sample results in a quenching of the Ti^3+^ center signals and the formation of surface bound O_2_^–^ radicals. In addition, the underlying oxidation process results in a change of the isotropic signal, which is observed even in the presence of adsorbed molecular oxygen. The latter observation may indicate that the underlying species are not directly located on the surface of the TiO_2_ particle network. For further analysis, a sample, which was previously exposed to oxygen gas at 298 K, was subsequently annealed at 493 K in vacuum (*p* < 10^–5^ mbar). After cooling to 77 K, the EPR spectrum (Figure 5c) clearly demonstrates that annealing annihilates the paramagnetic O_2_^–^ by their decomposition into diamagnetic products. The remaining resonance signal at *g* = 2.0028 indicates the stability of the underlying species inside the re-oxidized nanoparticle network (Figure 5c). In addition to the identical *g* factor values (Figure 5a,c), the microwave saturation behavior (Figure 5e) is also comparable to that of the signal prior to O_2_^–^ decomposition (Figure 5d). This suggests that the underlying point defects are not significantly affected by oxidative treatment even though changes of the line width and signal intensity indicate some impact of the treatment on these species as well. The lower thermal stability of the O_2_^–^ ions as compared to the electron center at *g* = 2.0028 can be utilized to isolate its signal by annealing to *T* = 473 K as exclusive resonance contribution for further analysis.

**Figure 6:**
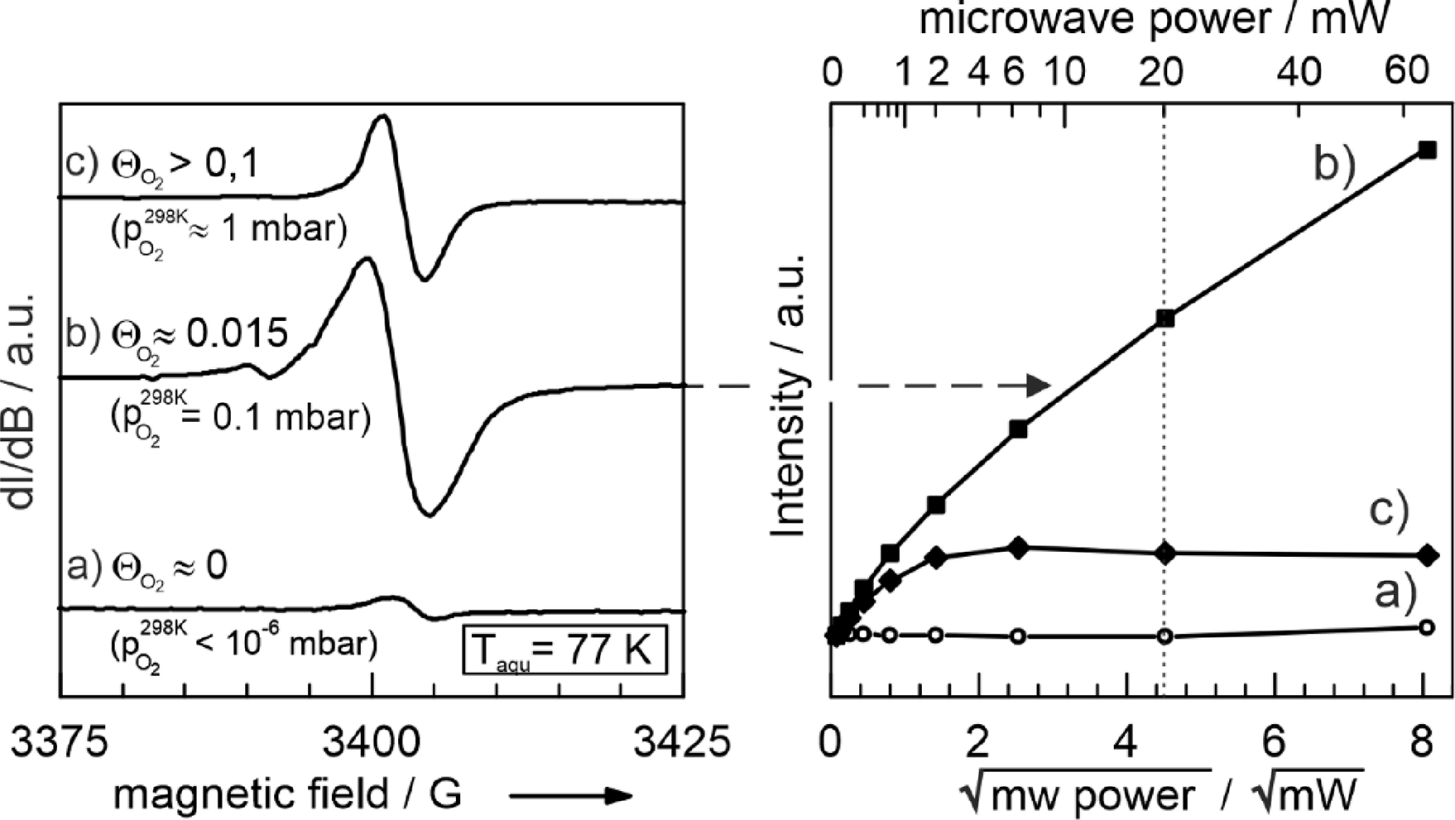
O_2_ coverage (Θ_O2_) dependent EPR signal of the electron center using a microwave power of 20 mW (left panel). The right panel displays the corresponding microwave power dependence of the electron center signal for different O_2_ coverages (Θ_O2_) as shown in the left panel.

In a next step, we varied the O_2_ pressures in the sample cell. The spectra for two additional pressures – in comparison the vacuum spectrum is shown in panel (c) – are shown in the left panel of Figure 6. All spectra exhibit a single isotropic signal at *g* = 2.0028, which differs significantly in intensity.

A systematic study for different oxygen pressures (see Figure S3, Supporting Information) reveals an EPR signal intensity maximum at about 0.1 mbar if using a microwave power of 200 μW. This pronounced dependence of the EPR intensity vanishes for measurements at very low microwave power (6.3 μW; Figure S4). These O_2_ coverage-dependent trends are attributed to changes of the spin lattice relaxation times as can be concluded from power saturation plots (right panel of Figure 6).

The independence of the signal intensity on oxygen pressure at low microwave power reveals a comparable number of paramagnetic centers. However, the observed intensity behavior at higher microwave powers cannot be attributed to one single type of paramagnetic defect alone. In addition to spin exchange effects at higher O_2_ coverages, we assume that there are at least two signal components with distinct microwave saturation properties, which comprise the total signal.

### Discussion of the Electron Center: Its Origin and O_2_ Adsorption-Dependent Properties

#### Electron Trapping at Carbon Impurities

As outlined above, the EPR resonance signals at *g* = 2.0018 and – after contact with O_2_ – at *g* = 2.0028 (Figure 3a) reveal a small *g*-tensor anisotropy with all components close to the free spin value. The EPR properties of the underlying spin center are very different from those of Ti^3+^ with their characteristic anisotropic symmetry.^8,59^

Although the materials characterized for this study were derived from high purity chemicals and synthesized in the gas phase, at high temperatures and in an O_2_ atmosphere, we investigated the hypothesis that the unintentional admixture of smallest amounts of carbon may be linked to the here reported EPR resonances. In TiO_2_ nanoparticle synthesis – irrespective of whether performed in the gas phase or in solution – carbon impurities cannot be excluded, despite the fact that carbon can be effectively removed by sample calcination in an oxygen atmosphere.^11,61,62^ As outlined in previous work^1^ and revealed by a quantitative analysis of FSP nanoparticle powders after oxidative annealing treatment here, the EPR center concentrations are in the range between 1 and 10 ppm (Table S1, Supporting Information). Depending on particle size, this corresponds to 0.02 (FSP TiO_2_ with *d*_av_ = 8.5 ± 2.5 nm) to 0.1 (CVS TiO_2_ with *d*_av_ = 12 ± 6 nm) spins per particle. (Corresponding concentrations are far below those of carbon impurities that would originate from the instantaneous surface contamination with hydrocarbons from air.^63^ Such contamination effects, however, represent an inevitable step during sample transfer from the sample cell/EPR tube into an analysis chamber for carbon analysis (*c* ≥ 1000 ppm)).

**Figure 7:**
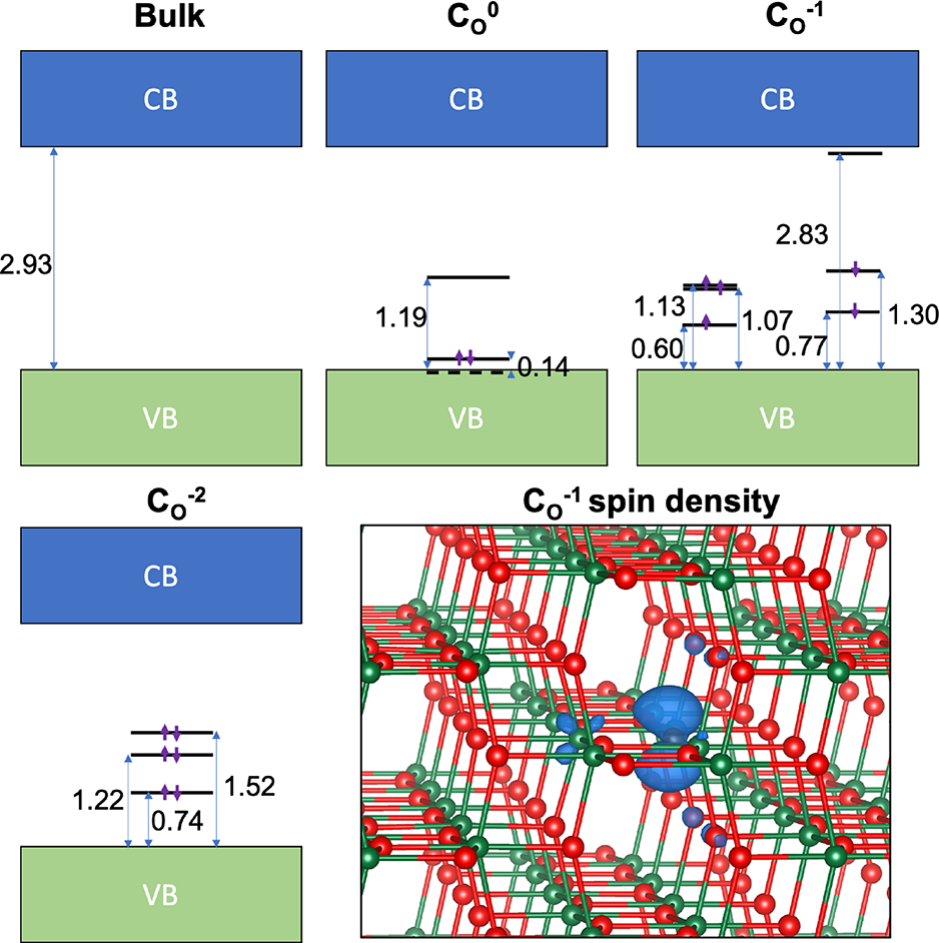
Predicted electronic structure of bulk anatase TiO_2_ bulk and C_O_ defects in *q* = 0, −1e, and −2e charge states. The spin density associated with the *q* = −1e defect is also shown which is primarily localized on C (2p) with a smaller contribution from neighboring Ti (3d) and O (2p).

Previous DFT calculations for carbon-related defects in TiO_2_ by Di Valentin *et al*.^30^ have shown that under oxygen-poor conditions, oxygen substitution by carbon (C_O_) inside the anionic sublattice of anatase is thermodynamically favorable, whereas in oxygen-rich conditions, interstitial carbon and carbon substituting for titanium are preferred. Since anatase nanoparticles are typically reduced to some extent, C_O_ is expected to form more readily in the nanoparticle networks studied in the present work (Figure 7). The C_O_ defect was predicted to introduce six C 2*p* levels in the anatase band gap. Since only four of these levels are occupied in the neutral (*q* = 0) state, this defect could present a prospective trap for electrons photoexcited into the conduction band. To further investigate this hypothesis, we revisit the C_O_ defect using a more advanced DFT approach specifically developed to model charge carrier trapping in TiO_2_ (see Methods for details).

The predicted electronic structures of the C_O_ defect in the *q* = 0, −1e, and −2e charge states are summarized in Figure 7. The neutral defect (C_O_^0^) has two doubly occupied C 2p levels. The first is a resonant state very close to the valence band maximum (VBM) with the second 0.14 eV above the VBM. The third unoccupied 2p level sits 1.19 eV above the VBM. The results for this defect are qualitatively similar to the previously reported results by Di Valentin *et al*.;^30^ however the C 2*p* levels are typically deeper (consistent with the fact that functional used here should suffer much less from self-interaction error) and a band gap in better agreement with experiments is obtained. The C_O_^–1^ defect is spin-polarized with five electrons distributed over five C 2p levels close to the VBM (within a range 0.6–1.3 eV) and a further unoccupied 2p level that sits just below the conduction band minimum. The spin density is mainly localized on the C atom in a 2p orbital but there is some hybridization with Ti 3d and O 2p orbitals on adjacent atoms. The fact that the spin density is primarily localized on C, which has small spin-orbit coupling, would be consistent with a *g* factor close to the free electron value. The thermodynamic charge transition level (CTL: the Fermi energy for which the formation energy of the neutral and negatively charged defects are equal) is 1.8 eV above the VBM. The C_O_^–2^ defect has six electrons occupying C 2p states in the range 0.7–1.5 eV above VBM with no other unoccupied states in the gap. The corresponding CTL between the −1 and −2 charge states is 2.2 eV above the VBM.

The CTLs for the C_O_ defects are deeper than those associated with the oxygen vacancies in anatase (which are close to the conduction band minimum). Therefore, in equilibrium, one would expect C_O_ defects to act as a double trap for electrons donated by oxygen vacancies, thereby reducing the intrinsic *n*-type carrier concentration. If the number of oxygen vacancies exceeds that of C_O_, one would expect almost all C_O_ defects to be in the diamagnetic *q* = −2e charge state. However, single photoionization of these defects or double photoionization followed by trapping of a single electron from the conduction band (both feasible with near-IR to visible light) would lead to transient formation of the spin polarized C_O_^–1^ defect. Another possibility to explain this apparent contradiction would be that the properties of C_O_ defects in the strained regions near nanoparticle necks could be sufficiently different to make the concentration of negatively charged C_O_ defects in equilibrium non-negligible.

As shown in Figure 3, the intensity of the EPR signals increases significantly upon adsorption of O_2_, which traps electrons as an electron acceptor. Related changes in the number of electrons (*n*-type carrier concentration) in TiO_2_ may shift the equilibrium between C_O_^–1^ and C_O_^–2^ states and could explain the EPR intensity increase and the slight shift in the *g* value. From previous experimental studies where we analyzed oxygen adsorption at different semiconductor metal oxide nanoparticle powders,^64^ we learned that the position of the O_2_/O_2_^–^ redox potential energy lies less than 0.3 eV below the bottom of the anatase TiO_2_ conduction band. This would also explain the absence of electron transfer from deep trap states to adsorbed O_2_.

#### Electron Center and Its O_2_ Adsorption-Dependent EPR Properties

Electron transfer from Ti^3+^ sites to O_2_ resulting in the formation of adsorbed O_2_^–^ can be effectively tracked by EPR spectroscopy.^61^ The electron transfer from the underlying shallow trap state to molecular O_2_ is enabled by the electron affinity of O_2_ with values between 0.3 and 0.44 eV. In turn, the lowest unoccupied molecular orbital (LUMO), that serves as the electron acceptor, is located slightly below the shallow trapped state of the reduced anatase. This explains spontaneous charging of the adsorbed O_2_ molecules.^65^ Compared to Ti^3+^ sites as shallow trap sites in the surface or subsurface region, the EPR signal of the electron center at *g* = 2.0028 is not quenched upon oxygen adsorption. Hence, electron transfer onto adsorbed oxygen molecules does not take place, which may be due to position of the C_O_^–1^ related states in the band gap (Figure 7).

**Figure 8:**
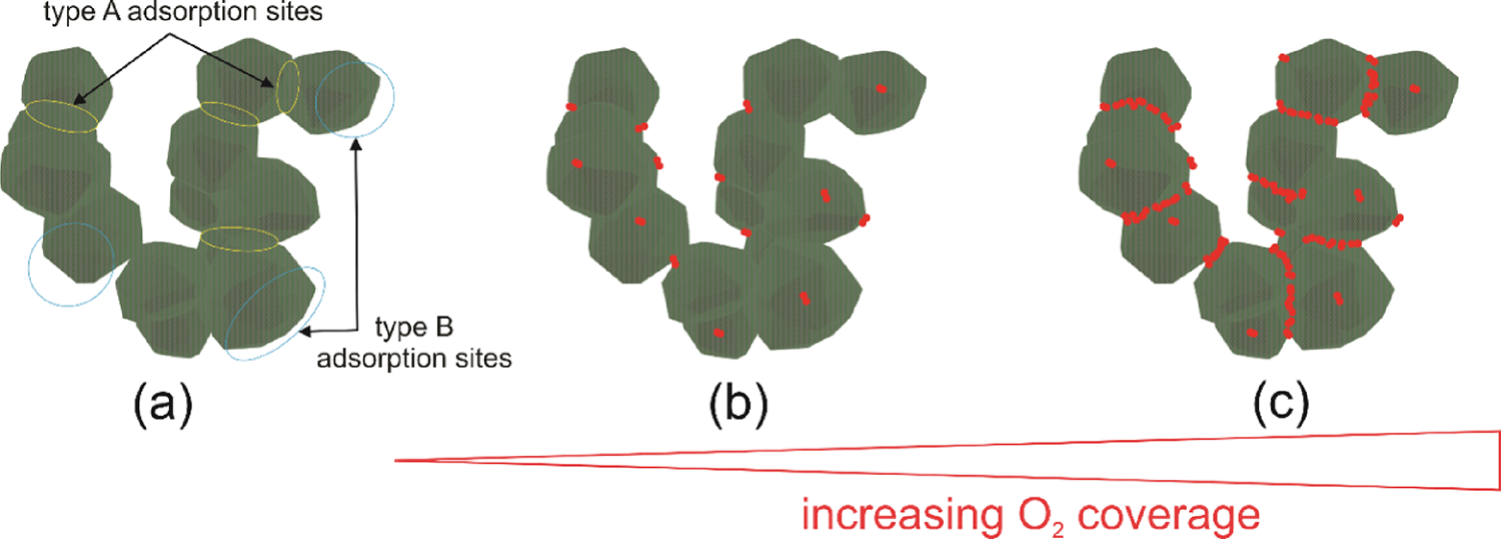
Schematic presentation of a section of a nanoparticle network (a) that exhibits qualitatively different O_2_ adsorption sites, namely, type A and type B. With increasing O_2_ coverage, we expect preferential O_2_ condensation in the neck region between adjacent grains (Figure 8c, type A), whereas less molecules adsorb at the B-type sites that are remote (Figure 8b).

The local concentration of O_2_ that is adsorbed in the vicinity of the defect hosting the unpaired electron determines the signal intensity. It can either lead to an intensity increase (shortening of the relaxation time *T*_1_ by spin exchange and, thus, diminished microwave power saturation) or to its depletion (quenching at defect sites where O_2_ preferentially accumulates to form adsorbate clusters). The rise and fall of the relaxation-dependent intensities with O_2_ coverage (Figure 6 and Figure S3) can be rationalized by a model that assumes the presence of two types of spin centers with identical local environments but different locations inside the heterogeneous nanoparticle network. With respect to the first coordination sphere, the electrons experience a spherically symmetric electric field distribution. With respect to their location inside the nanoparticle network, the two types of defects are different (Figure 8). They can exist in regions where sinter necks interconnect adjacent nanoparticles (Figure 8, type A adsorption sites) or they can be located in parts of the nanoparticle network that are remote from such necks, i.e., in the surface or subsurface region of the individual particles (Figure 8, type B adsorption sites).

Impinging at the nanoparticle network surface, O_2_ molecules experience a strong heterogeneity in terms of adsorption energetics. Nanoscale capillary condensation in the neck region has been explored on TiO_2_ fine powders.^66,67^ A recent study investigated supported WO_3_ nanoparticles with scanning tunneling microscopy and spectroscopy (STM-STS)^68^ and revealed preferential molecule adsorption in the neck region of the nanoparticles, which would correspond to type A adsorption sites (Figure 8a). The authors also succeeded in spatially resolving electronic responses from different particle regions in vacuum and after exposure to O_2_. As a result, the study provides strong evidence for preferential reduction in the neck region^51,61^ connecting adjacent grains and, furthermore, that region facilitates increased O_2_ adsorption.

#### Trapping of Carbon Impurities

**Figure 9:**
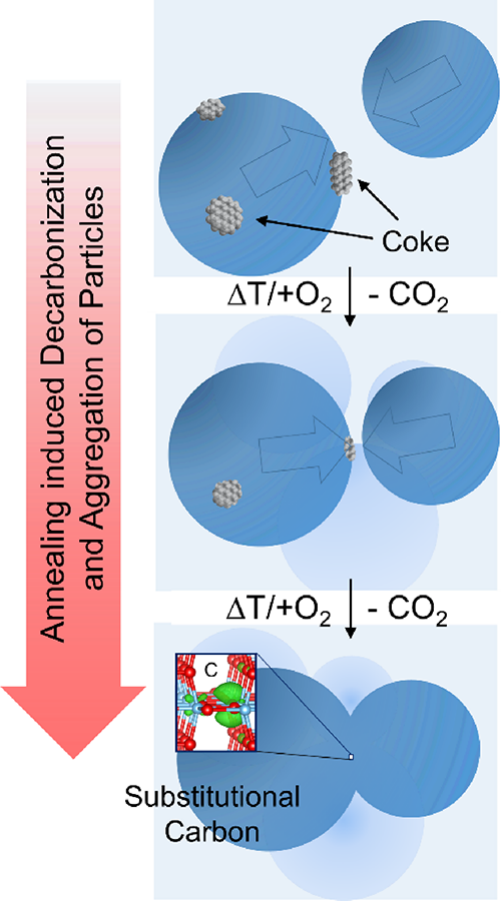
Scheme that illustrates how synthesis and processing induced particle coagulation converts surface carbon impurities into dopants of the host lattice. The different stages from the top to the bottom should also indicate the progressive elimination of carbon by combustion treatment in O_2_ down to the level of a few ppm.

The computational analysis outlined in the discussion section A provides a sound explanation for the EPR resonances in slightly oxygen deficient TiO_2_ nanoparticle networks. Carbon at the lowest concentrations can substitute positions in the oxygen sublattice to form C_O_^–1^ sites. At this point, the question arises why hard aggregate formation and particle necking as illustrated by Figure 2 and the rationalization of the O_2_ adsorption-dependent EPR properties (Figure 6) would promote carbon incorporation into the TiO_2_ lattice. A simple and intuitively comprehensible answer would be that during all the dynamic processes that occur during oxidative carbon removal (Figure 9, top panel) at elevated temperatures, the attachment and fusion of the particles may lead to trapping of residual traces of carbon inside the contact region, which converts from surface structures into bulk species (Figure 9, bottom panel). Such particle attachment and necking can occur during the particle formation and aggregation process in the flame (FSP, Figures 1a–c and 2c) because of hydration/dehydration treatment of pre-cleaned MOCVS nanoparticle powders (Figure 2a), which may still contain traces of carbon at the particle surfaces or the controlled decomposition and crystallization of a carbon-rich titania aerogel (Figure 2b).

## Conclusions

TiO_2_ nanoparticle synthesis and associated powder processing involves particle coagulation and aggregation (Figures 1 and 2). This is particularly true for solvent-based approaches and for evacuation steps of previously hydrated materials (Figure 2b). During coagulation and neck formation between particles, surface impurities such as carbonaceous species become entrapped between particles and convert to substitutional carbon in the anionic sublattice of TiO_2_.

Whereas the stabilization of electrons in the d orbitals of Ti atoms is strongly favored in pure and carbon-free TiO_2_ materials as opposed to the trapping of unpaired electrons inside oxygen vacancies, substitutional carbon, already with concentrations as low as a few ppm, traps unpaired electrons and offers a way to localize the spin in the TiO_2_ lattice. The here-discussed EPR properties of the spin center and its O_2_ adsorption induced changes correspond to those of localized and isolated unpaired electrons in defects or radicals^69,70^ rather than to the O_2_ adsorption-induced localization of conduction band electrons as suggested in previous studies.^25−27^

## Supporting Information Available

X-ray diffraction pattern, particle size distribution plots, O_2_ coverage-dependent EPR signal intensity changes, and an estimate of trapped carbon impurities (PDF)

## References

[ref1] Baumann S. O.; Elser M. J.; Auer M.; Bernardi J.; Hüsing N.; Diwald O. Solid-Solid Interface Formation in TiO_2_ Nanoparticle Networks. Langmuir 2011, 27, 1946–1953. 10.1021/la104213d. 21265546

[ref2] Tuller H. L.; Bishop S. R. Point Defects in Oxides: Tailoring Materials Through Defect Engineering. Annu. Rev. Mater. Res. 2011, 41, 369–398. 10.1146/annurev-matsci-062910-100442.

[ref3] Giordano L.; Akkiraju K.; Jacobs R.; Vivona D.; Morgan D.; Shao-Horn Y. Electronic Structure-Based Descriptors for Oxide Properties and Functions. Acc. Chem. Res. 2022, 55, 298–308. 10.1021/acs.accounts.1c00509. 35050573

[ref4] Diwald O.; Berger T., Eds.; Metal Oxide Nanoparticles: Formation, Functional Properties, and Interfaces: John Wiley & Sons Ltd, Hoboken, 2022. 10.1002/9781119436782.

[ref5] Berger T.; Diwald O. Defects in Metal Oxide Nanoparticle Powders: in Defects at Oxide Surfaces Jupille J., Thornton G., Eds.; Springer Series in Surface Sciences, vol 58, Springer: Cham, 2015; pp. 273–301. 10.1007/978-3-319-14367-5_9

[ref6] Shahiduzzaman M.; Ismail Hossain M.; Otani S.; Wang L.; Umezu S.; Kaneko T.; Iwamori S.; Tomita K.; Hong Tsang Y.; Akhtaruzzaman M.; et al. Low-Temperature Treated Anatase TiO_2_ Nanophotonic-Structured Contact Design for Efficient Triple-Cation Perovskite Solar Cells. Chem. Eng. J. 2021, 426, 131831 10.1016/j.cej.2021.131831.

[ref7] Siedl N.; Elser M. J.; Bernardi J.; Diwald O. Functional Interfaces in Pure and Blended Oxide Nanoparticle Networks: Recombination versus Separation of Photogenerated Charges. J. Phys. Chem. C 2009, 113, 15792–15795. 10.1021/jp906368f.

[ref8] Chiesa M.; Paganini M. C.; Livraghi S.; Giamello E. Charge Trapping in TiO_2_ Polymorphs as Seen by Electron Paramagnetic Resonance Spectroscopy. Phys. Chem. Chem. Phys. 2013, 15, 9435–9447. 10.1039/c3cp50658d. 23695705

[ref9] Czoska A. M.; Livraghi S.; Chiesa M.; Giamello E.; Agnoli S.; Granozzi G.; Finazzi E.; Di Valentiny C.; Pacchioni G. The Nature of Defects in Fluorine-Doped TiO_2_. J. Phys. Chem. C 2008, 112, 8951–8956. 10.1021/jp8004184.

[ref10] Chiesa M.; Giamello E. On the Role and Applications of Electron Magnetic Resonance Techniques in Surface Chemistry and Heterogeneous Catalysis. Catal. Lett. 2021, 151, 3417–3436. 10.1007/s10562-021-03576-x.

[ref11] Paganini M. C.; Chiesa M.; Giamello E.; Coluccia S.; Martra G.; Murphy D. M.; Pacchioni G. Colour Centres at the Surface of Alkali-Earth Oxides. A New Hypothesis on the Location of Surface Electron Traps. Surf. Sci. 1999, 421, 246–262. 10.1016/S0039-6028(98)00795-X.

[ref12] Pinarello G.; Pisani C.; D’Ercole A.; Chiesa M.; Paganini M. C.; Giamello E.; Diwald O. O^–^ Radical Ions on MgO as a Tool to Unravel Structure and Location of Ionic Vacancies at the Surface of Oxides: A Coupled Experimental and Theoretical Investigation. Surf. Sci. 2001, 494, 95–110. 10.1016/S0039-6028(01)01374-7.

[ref13] Brant A. T.; Giles N. C.; Halliburton L. E. Hydrogen Donors and Ti^3+^ Ions in Reduced TiO_2_ Crystals. J. Appl. Phys. 2011, 110, 53714 10.1063/1.3630964.

[ref14] Brant A. T.; Giles N. C.; Yang S.; Sarker M. A. R.; Watauchi S.; Nagao M.; Tanaka I.; Tryk D. A.; Manivannan A.; Halliburton L. E. Ground State of the Singly Ionized Oxygen Vacancy in Rutile TiO_2_. J. Appl. Phys. 2013, 114, 113702 10.1063/1.4819805.

[ref15] Yang S.; Halliburton L. E.; Manivannan A.; Bunton P. H.; Baker D. B.; Klemm M.; Horn S.; Fujishima A. Photoinduced Electron Paramagnetic Resonance Study of Electron Traps in TiO_2_ Crystals: Oxygen Vacancies and Ti^3+^ Ions. Appl. Phys. Lett. 2009, 94, 162114 10.1063/1.3124656.

[ref16] Di Valentin C.; Pacchioni G. Spectroscopic Properties of Doped and Defective Semiconducting Oxides from Hybrid Density Functional Calculations. Acc. Chem. Res. 2014, 47, 3233–3241. 10.1021/ar4002944. 24828320

[ref17] Chiesa M.; Paganini M. C.; Giamello E.; Murphy D. M.; Di Valentin C.; Pacchioni G. Excess Electrons Stabilized on Ionic Oxide Surfaces. Acc. Chem. Res. 2006, 39, 861–867. 10.1021/ar068144r. 17115726

[ref18] Quirk J. A.; Lazarov V. K.; McKenna K. P. First-Principles Modeling of Oxygen-Deficient Anatase TiO_2_ Nanoparticles. J. Phys. Chem. C 2020, 124, 23637–23647. 10.1021/acs.jpcc.0c06052.

[ref19] Di Valentin C.; Pacchioni G.; Selloni A. Reduced and n-type Doped TiO_2_: Nature of Ti^3+^ Species. J. Phys. Chem. C 2009, 113, 20543–20552. 10.1021/jp9061797.

[ref20] Nakamura I.; Negishi N.; Kutsuna S.; Ihara T.; Sugihara S.; Takeuchi K. Role of Oxygen Vacancy in the Plasma-Treated TiO_2_ Photocatalyst with Visible Light Activity for NO Removal. J. Mol. Catal. A 2000, 161, 205–212. 10.1016/S1381-1169(00)00362-9.

[ref21] Reyes-Garcia E. A.; Sun Y.; Reyes-Gil K. R.; Raftery D. Solid-State NMR and EPR Analysis of Carbon-Doped Titanium Dioxide Photocatalysts (TiO_2-x_C_x_). Solid State Nucl. Magn. Reson. 2009, 35, 74–81. 10.1016/j.ssnmr.2009.02.004. 19307109

[ref22] Che M.; Védrine J. C., Eds.; Characterization of solid materials and heterogeneous catalysts*;* Wiley-VCH Verlag GmbH & Co. KGaA: Weinheim, 2012.

[ref23] Acharya S.; Torgersen J.; Kim Y.; Park J.; Schindler P.; Dadlani A. L.; Winterkorn M.; Xu S.; Walch S. P.; Usui T.; et al. Self-limiting Atomic Layer Deposition of Barium Oxide and Barium Titanate Thin Films Using a Novel Pyrrole Based Precursor. J. Mater. Chem. C 2016, 4, 1945–1952. 10.1039/C5TC03561A.

[ref24] Li Y.; Hwang D.-S.; Lee N. H.; Kim S.-J. Synthesis and Characterization of Carbon-Doped Titania as an Artificial Solar Light Sensitive Photocatalyst. Chem. Phys. Lett. 2005, 404, 25–29. 10.1016/j.cplett.2005.01.062.

[ref25] Serwicka E. Localization of Conduction Band Electrons in Polycrystalline TiO_2_ Studied by ESR. Z. Naturfor. Section A J. Phys. Sci. 1981, 36, 226–232.

[ref26] Serwicka E.; Schlierkamp M. W.; Schindler R. N. Localization of Conduction-Band Electrons in Polycrystalline TiO_2_ Studied by Electron-Spin-Resonance. Z. Naturfor. 1981, 36, 226 10.1515/zna-1981-0305.

[ref27] Serwicka E. M. ESR of polycrystalline titania reduced at room temperature with H atoms. Z. Phys. Chem. 1990, 166, 249–252. 10.1524/zpch.1990.166.Part_2.249.

[ref28] Schneider J.; Matsuoka M.; Takeuchi M.; Zhang J.; Horiuchi Y.; Anpo M.; Bahnemann D. W. Understanding TiO_2_ Photocatalysis: Mechanisms and Materials. Chem. Rev. 2014, 114, 9919–9986. 10.1021/cr5001892. 25234429

[ref29] Zhang T.; Low J.; Yu J.; Tyryshkin A. M.; Mikmeková E.; Asefa T. A Blinking Mesoporous TiO_2–x_ Composed of Nanosized Anatase with Unusually Long-Lived Trapped Charge Carriers. Angew. Chem., Int. Ed. 2020, 59, 15000–15007. 10.1002/anie.202005143. 32445242

[ref30] Di Valentin C.; Pacchioni G.; Selloni A. Theory of Carbon Doping of Titanium Dioxide. Chem. Mater. 2005, 17, 6656–6665. 10.1021/cm051921h.

[ref31] Guskos N.; Anagnostakis E. A.; Zolnierkiewicz G.; Typek J.; Biedunkiewicz A.; Guskos A.; Berczynski P. Effect of Annealing on EPR Spectra of Ti-Si-C-N Samples. Mater Sci-Pol. 2012, 30, 23–31. 10.2478/s13536-012-0004-5.

[ref32] Guskos N.; Bodziony T.; Maryniak M.; Typek J.; Biedunkiewicz A. Paramagnetic Centers in Nanocrystalline TiC/C System. J. Alloys Compd. 2008, 455, 52–54. 10.1016/j.jallcom.2007.01.071.

[ref33] Dai H.; Wong E. W.; Lu Y. Z.; Fan S.; Lieber C. M. Synthesis and Characterization of Carbide Nanorods. Nature 1995, 375, 769–772. 10.1038/375769a0.

[ref34] Miller D. N.; Azad A. K.; Delpouve H.; Quazuguel L.; Zhou J.; Sinha A.; Wormald P.; Irvine J. T. S. Studies on the Crystal Structure, Magnetic and Conductivity Properties of Titanium Oxycarbide Solid Solution (TiO_1–x_ C _x_ ). J. Mater. Chem. A 2016, 4, 5730–5736. 10.1039/C6TA00042H.

[ref35] Minnekhanov A. A.; Deygen D. A.; Konstantinova E. A.; Vorontsov A. S.; Kashkarov P. K. Paramagnetic Properties of Carbon-doped Titanium Dioxide. Nanoscale Res. Lett. 2012, 7, 333 10.1186/1556-276X-7-333. 22720786 PMC3406989

[ref36] Livraghi S.; Corazzari I.; Paganini M. C.; Ceccone G.; Giamello E.; Fubini B.; Fenoglio I. Decreasing the Oxidative Potential of TiO_2_ Nanoparticles Through Modification of the Surface with Carbon: A New Strategy for the Production of Safe UV Filters. Chem. Commun. 2010, 46, 8478–8480. 10.1039/c0cc02537b. 20938530

[ref37] Schneider J., Ed. Photocatalysis: Fundamentals and perspectives; RSC energy and environment series; Royal Society of Chemistry, RSC Publ, Cambridge UK, 2015.

[ref38] Elser M. J.; Berger T.; Brandhuber D.; Bernardi J.; Diwald O.; Knözinger E. Particles Coming Together: Electron Centers in Adjoined TiO_2_ Nanocrystals. J. Phys. Chem. B 2006, 110, 7605–7608. 10.1021/jp0607465. 16610847

[ref39] Schneider J.; Ziegler A.; Zickler G. A.; Dzik P.; Berger T.; Diwald O. TiO_2_ Anatase and Rutile Grains and the Effect of Particle Printing on Porphyrin Adsorption. Surf. Sci. 2022, 722, 122083 10.1016/j.susc.2022.122083.

[ref40] Niedermaier M.; Schwab T.; Diwald O. Nanoparticle Synthesis in the Gas Phase, in: Metal Oxide Nanoparticles: Formation, Functional Properties, and Interfaces*,* Diwald O.; Berger T., Eds.; John Wiley & Sons Ltd, Hoboken, 2022. 10.1002/9781119436782.ch3

[ref41] Guidon M.; Hutter J.; VandeVondele J. Robust Periodic Hartree-Fock Exchange for Large-Scale Simulations Using Gaussian Basis Sets. J. Chem. Theory Comput. 2009, 5, 3010–3021. 10.1021/ct900494g. 26609981

[ref42] Elmaslmane A. R.; Watkins M. B.; McKenna K. P. First-Principles Modeling of Polaron Formation in TiO_2_ Polymorphs. J. Chem. Theory Comput. 2018, 14, 3740–3751. 10.1021/acs.jctc.8b00199. 29874462

[ref43] Elmaslmane A. R.; Wetherell J.; Hodgson M. J. P.; McKenna K. P.; Godby R. W. Accuracy of Electron Densities Obtained via Koopmans-Compliant Hybrid Functionals. Phys. Rev. Materials 2018, 2, 040801 10.1103/PhysRevMaterials.2.040801.

[ref44] VandeVondele J.; Krack M.; Mohamed F.; Parrinello M.; Chassaing T.; Hutter J. Quickstep: Fast and Accurate Density Functional Calculations Using a Mixed Gaussian and Plane Waves Approach. Comput. Phys. Commun. 2005, 167, 103–128. 10.1016/j.cpc.2004.12.014.

[ref45] VandeVondele J.; Hutter J. Gaussian Basis Sets for Accurate Calculations on Molecular Systems in Gas and Condensed Phases. J. Chem. Phys. 2007, 127, 114105 10.1063/1.2770708. 17887826

[ref46] Goedecker S.; Teter M.; Hutter J. Separable Dual-Space Gaussian Pseudopotentials. Phys. Rev. B 1996, 54, 1703–1710. 10.1103/PhysRevB.54.1703. 9986014

[ref47] Hartwigsen C.; Goedecker S.; Hutter J. Relativistic Separable Dual-Space Gaussian Pseudopotentials from H to Rn. Phys. Rev. B 1998, 58, 3641–3662. 10.1103/PhysRevB.58.3641. 9986014

[ref48] Krack M. Pseudopotentials for H to Kr Optimized for Gradient-Corrected Exchange-Correlation Functionals. Theor. Chem. Acc. 2005, 114, 145–152. 10.1007/s00214-005-0655-y.

[ref49] Momma K.; Izumi F. VESTA 3 for Three-Dimensional Visualization of Crystal, Volumetric and Morphology Data. J. Appl. Crystallogr. 2011, 44, 1272–1276. 10.1107/S0021889811038970.

[ref50] Chang H.; Kim S. J.; Jang H. D.; Choi J. W. Synthetic Routes for Titania Nanoparticles in the Flame Spray Pyrolysis. Colloids Surf., A Physicochem. Eng. Asp. 2008, 313-314, 282–287. 10.1016/j.colsurfa.2007.04.111.

[ref51] Kocsis K.; Niedermaier M.; Bernardi J.; Berger T.; Diwald O. Changing Interfaces: Photoluminescent ZnO Nanoparticle Powders in Different Aqueous Environments. Surf. Sci. 2016, 652, 253–260. 10.1016/j.susc.2016.02.019. 32903287 PMC7116034

[ref52] Carl K.; Dikhoff J. A. M.; Eckenbach W.; Junginger H. G. On the Limits of the Filter Concept for Color TV Screens. J. Electrochem. Soc. 1981, 128, 2395–2401. 10.1149/1.2127258.

[ref53] Kho Y. K.; Iwase A.; Teoh W. Y.; Mädler L.; Kudo A.; Amal R. Photocatalytic H_2_ Evolution over TiO_2_ Nanoparticles. The Synergistic Effect of Anatase and Rutile. J. Phys. Chem. C 2010, 114, 2821–2829. 10.1021/jp910810r.

[ref54] Baumann S. O.; Schneider J.; Sternig A.; Thomele D.; Stankic S.; Berger T.; Grönbeck H.; Diwald O. Size Effects in MgO Cube Dissolution. Langmuir 2015, 31, 2770–2776. 10.1021/la504651v. 25668706

[ref55] Neige E.; Diwald O. Paramagnetic Electron Centers in BaTiO_3_ Nanoparticle Powders. Phys. Chem. Chem. Phys. 2021, 23, 12881–12888. 10.1039/D1CP01128F. 34075975

[ref56] Chowdhury I.; Walker S. L.; Mylon S. E. Aggregate Morphology of Nano-TiO_2_: Role of Primary Particle Size, Solution Chemistry, and Organic Matter. Environ. Sci.: Process. Impacts 2013, 15, 275–282. 10.1039/C2EM30680H. 24592445

[ref57] Berger T.; Diwald O. Traps and Interfaces in Photocatalysis: Model Studies on TiO_2_ Particle Systems. in: Photocatalysis; Schneider J., Bahnemann D., Ye J., Li Puma G., Dionysiou D. D., Eds.; RSC energy and environment series; Royal Society of Chemistry: Cambridge UK, 2016; pp. 185–217.

[ref58] Livraghi S.; Maurelli S.; Paganini M. C.; Chiesa M.; Giamello E. Probing the local environment of Ti^3+^ ions in TiO_2_ (rutile) by ^17^O HYSCORE. Angew. Chem. - Int. Ed. 2011, 50, 8038–8040. 10.1002/anie.201100531. 21744443

[ref59] Livraghi S.; Chiesa M.; Paganini M. C.; Giamello E. On the Nature of Reduced States in Titanium Dioxide As Monitored by Electron Paramagnetic Resonance I: The Anatase Case. J. Phys. Chem. C 2011, 115, 25413–25421. 10.1021/jp209075m.

[ref60] Berger T.; Sterrer M.; Diwald O.; Knözinger E.; Panayotov D.; Thompson T. L.; Yates J. T. Jr. Light-Induced Charge Separation in Anatase TiO_2_ Particles. J. Phys. Chem. B 2005, 109, 6061–6068. 10.1021/jp0404293. 16851666

[ref61] Elser M. J.; Diwald O. Facilitated Lattice Oxygen Depletion in Consolidated TiO_2_ Nanocrystal Ensembles: A Quantitative Spectroscopic O_2_ Adsorption Study. J. Phys. Chem. C 2012, 116, 2896–2903. 10.1021/jp208707p.

[ref62] Advances in Electronics and Electron Physics Volume 65; Elsevier, Eds. Marton L., Marton C., Smithonian Institution: Washington DC. 1985.

[ref63] Zubkov T.; Stahl D.; Thompson T. L.; Panayotov D.; Diwald O.; Yates J. T. Jr. Ultraviolet Light-Induced Hydrophilicity Effect on TiO_2_(110) (1×1). Dominant Role of the Photooxidation of Adsorbed Hydrocarbons Causing Wetting by Water Droplets. J. Phys. Chem. B 2005, 109, 15454–15462. 10.1021/jp058101c. 16852960

[ref64] Siedl N.; Baumann S. O.; Elser M. J.; Diwald O. Particle Networks from Powder Mixtures: Generation of TiO_2_-SnO_2_ Heterojunctions via Surface Charge-Induced Heteroaggregation. J. Phys. Chem. C 2012, 116, 22967–22973. 10.1021/jp307737s. PMC3558020 23378867

[ref65] Setvin M.; Hulva J.; Parkinson G. S.; Schmid M.; Diebold U. Electron Transfer Between Anatase TiO_2_ and an O_2_ Molecule Directly Observed by Atomic Force Microscopy. Proc. Natl. Acad. Sci. U. S. A. 2017, 114, E2556–E2562. 10.1073/pnas.1618723114. 28289217 PMC5380104

[ref66] Kim S.; Ehrman S. H. Capillary Condensation onto Titania (TiO_2_) Nanoparticle Agglomerates. Langmuir 2007, 23, 2497–2504. 10.1021/la062456l. 17243733

[ref67] Kim S.; Ehrman S. H. Grand Canonical Monte Carlo Simulation Study of Capillary Condensation between Nanoparticles. J. Chem. Phys. 2007, 127, 134702 10.1063/1.2786087. 17919038

[ref68] Ottaviano L.; Maccallini E.; Santucci S. Visualisation of the Preferential Adsorption Sites of Oxygen onto WO_3_ Nano-Particles. Surf. Sci. 2001, 492, L700–L704. 10.1016/S0039-6028(01)01440-6.

[ref69] Chiesa M.; Amato G.; Boarino L.; Garrone E.; Geobaldo F.; Giamello E. Reversible Insulator-to-Metal Transition in p+–Type Mesoporous Silicon Induced by the Adsorption of Ammonia. Angew. Chem., Int. Ed. 2003, 42, 5032–5035. 10.1002/anie.200352114. 14595623

[ref70] Brustolon M.; Giamello E. Electron Paramagnetic Resonance: A Practitioner’s Toolkit; John Wiley & Sons, Inc. Hoboken, N.J.; 2009.

