# Supplementary material for: On the Importance of Nanoparticle Necks and Carbon Impurities for Charge Trapping in TiO2
Source: J Phys Chem C Nanomater Interfaces. 2023 May 3;127(18):8778–87. doi: 10.1021/acs.jpcc.3c00430 (PMC10184168; doi:10.1021/acs.jpcc.3c00430)
Supplement: Supplementary file 1 — jp3c00430_si_001.pdf [file jp3c00430_si_001.pdf]

Supporting Information

On the Importance of Nanoparticle Necks and Carbon Impurities for Charge Trapping in TiO_2

Michael J. Elser[1], Ellie Neige[2], Thomas Berger[2], Mario Chiesa[3],

Elio Giamello[3], Keith McKenna[4], Thomas Risse[5], Oliver Diwald [2]**

keith.mckenna@york.ac.uk; oliver.diwald@plus.ac.at

[1] Institute of Particle Technology (LFG), Friedrich-Alexander-Universität Erlangen-Nürnberg, Cauerstraße 4, Erlangen, 91058, Germany

[2] Department of Chemistry and Physics of Materials, Paris-Lodron Universität Salzburg, Jakob-Haringerstrasse 2a, 5020 Salzburg, Austria

[3] Department of Chemistry and NIS Centre, University of Torino, via Giuria 7, I-10125 Torino, Italy

[4] School of Physics, Engineering and Technology, University of York, Heslington, YO10 5DD York, United Kingdom

[5] Institut für Chemie und Biochemie, Freie Universität Berlin, Arnimallee 22, 14195 Berlin, Germany

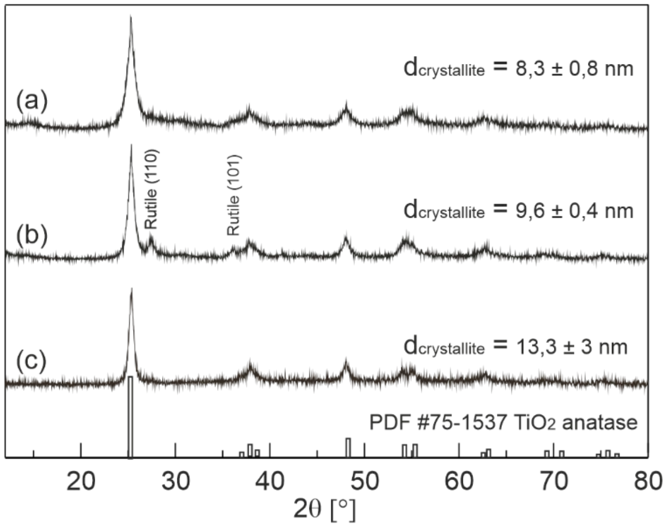

Figure S1: XRD pattern acquired on FSP synthesized TiO_2 nanoparticle powders before (a) and after (b) thermal processing at T = 873 K. For comparison, trace c shows the XRD pattern of phase pure TiO_2 anatase nanoparticles that were produced by metal organic chemical vapor deposition (MOCVS) and subjected to an identical thermal activation procedure as the sample related to the pattern (b) thereafter.

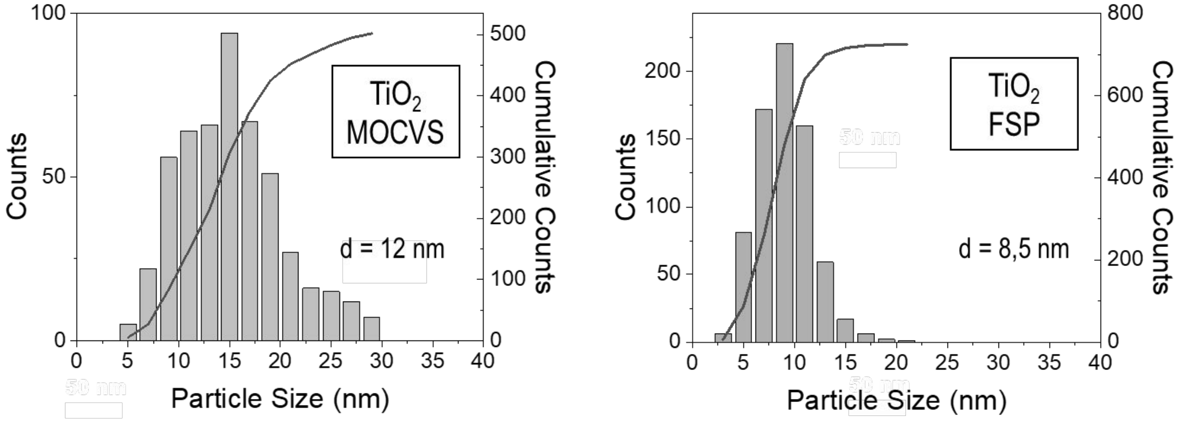

Figure S2: particle size distribution of TiO_2 nanoparticles synthesized by metal organic chemical vapor synthesis (MOCVS) (a) and flame spray pyrolysis (FSP) (b) after annealing in alternating vacuum/O_2 atmosphere at T = 873 K.

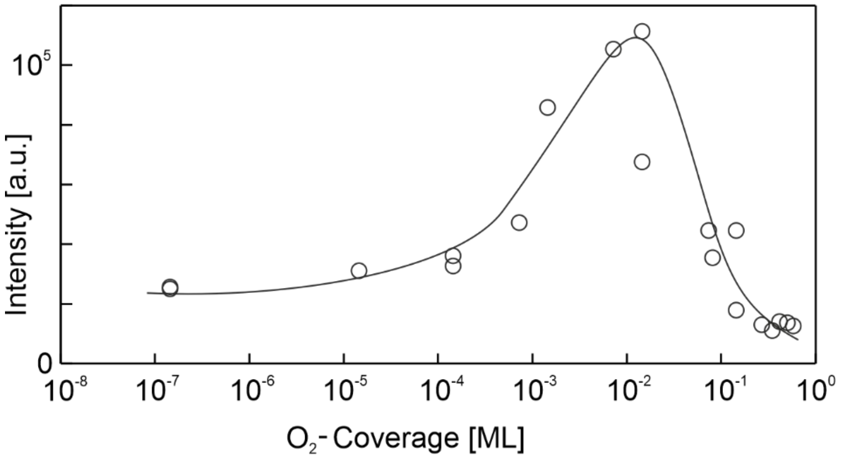

Figure S3: Signal intensity changes as a function of O_2 pressure applied at room temperature prior to cooling to T = 77 K for EPR spectrum

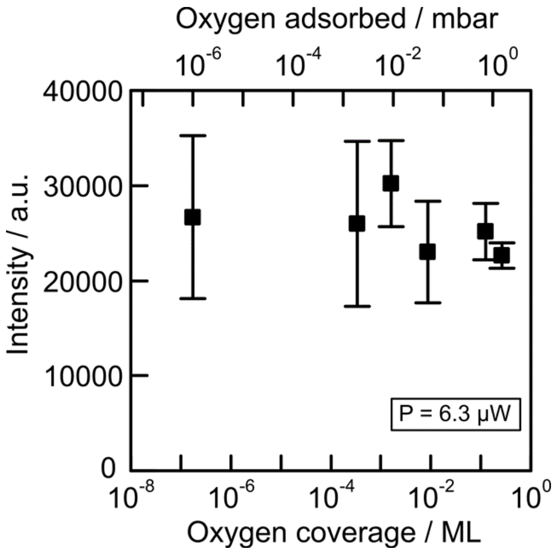

acquisition. The microwave power used was 200 μW (30 dB).

Figure S4: EPR signal intensity of the electron center as a function of oxygen coverage and measured at a microwave power of 6.3 μW.

Table S1: Average TiO_2 nanoparticle diameters and estimated concentration of paramagnetic defects.

sample	Particle size (nm)	Particle mass (kg)	Number of [TiO_2] formula units per particle	From spin counting (Bruker-Xenon software): Paramagnetic defects per particle	Estimated concentrati on of defects (ppm)
TiO2 CVS	12±6	$3.5\cdot10^{-21}$	$2.6\cdot10^{4}$	0.1 (1/10)	4±2
TiO2 FSP	8.5±2.5	$3.2\cdot10^{-21}$	$9.4\cdot10^{3}$	0.02 (1/50)	2±1
